# Quantitative dual-energy CT material decomposition of holmium microspheres: local concentration determination evaluated in phantoms and a rabbit tumor model

**DOI:** 10.1007/s00330-020-07092-1

**Published:** 2020-08-07

**Authors:** Ralf Gutjahr, Robbert C. Bakker, Feiko Tiessens, Sebastiaan A. van Nimwegen, Bernhard Schmidt, Johannes Frank Wilhelmus Nijsen

**Affiliations:** 1 Computed Tomography, Siemens Healthcare GmbH, Forchheim, Germany; 2 CAMP, Technical University of Munich, Munich, Germany; 3 Department of Radiology and Nuclear Medicine, University Medical Center Utrecht, Utrecht, The Netherlands; 4 Department of Oral and Maxillofacial Surgery, University Medical Center Utrecht, Utrecht, The Netherlands; 5 R&D Imaging & Software, Quirem Medical BV, Deventer, The Netherlands; 6 Department of Clinical Sciences of Companion Animals, Faculty of Veterinary Medicine, Utrecht University, Utrecht, The Netherlands; 7 Department of Medical Imaging, Radboudumc, Geert Grooteplein-Zuid 10, 6525 GA Nijmegen, The Netherlands

**Keywords:** Tomography, X-Ray computed, Contrast media, Holmium, Microspheres

## Abstract

**Objectives:**

The purpose of this study was to assess the feasibility of dual-energy CT-based material decomposition using dual-X-ray spectra information to determine local concentrations of holmium microspheres in phantoms and in an animal model.

**Materials and methods:**

A spectral calibration phantom with a solution containing 10 mg/mL holmium and various tube settings was scanned using a third-generation dual-energy CT scanner to depict an energy-dependent and material-dependent enhancement vectors. A serial dilution of holmium (microspheres) was quantified by spectral material decomposition and compared with known holmium concentrations. Subsequently, the feasibility of the spectral material decomposition was demonstrated in situ in three euthanized rabbits with injected (radioactive) holmium microspheres.

**Results:**

The measured CT values of the holmium solutions scale linearly to all measured concentrations and tube settings (*R*^2^ = 1.00). Material decomposition based on CT acquisitions using the tube voltage combinations of 80/150 Sn kV or 100/150 Sn kV allow the most accurate quantifications for concentrations down to 0.125 mg/mL holmium.

**Conclusion:**

Dual-energy CT facilitates image-based material decomposition to detect and quantify holmium microspheres in phantoms and rabbits.

**Key Points:**

*• Quantification of holmium concentrations based on dual-energy CT is obtained with good accuracy.*

*• The optimal tube-voltage pairs for quantifying holmium were 80/150 Sn kV and 100/150 Sn kV using a third-generation dual-source CT system.*

*• Quantification of accumulated holmium facilitates the assessment of local dosimetry for radiation therapies.*

## Introduction

In the 1970s and 1980s, dual-energy CT (DECT) technology demonstrated improved tissue characterization; however, the technique was not widely applied due to limitations like noise in low-kilo voltage (kV) images, acquisition time, and image registration difficulties [1–5]. Nowadays, DECT technology is clinically established as a result of fast technological developments, such as detectors with fully integrated electronics minimizing electronic noise, improved spectral separation using optimized beam pre-filtration, increased scan speed, and improved post processing techniques [6–11].

DECT uses two effective X-ray spectra, either generated by a single X-ray tube switching between two different X-ray spectra (kV-switch CT), by using two separated X-ray tubes applying two different voltages (dual-source CT, DSCT), or by resolving an incident spectrum at the scanner (dual-layer CT or photon-counting detector CT). Further image processing enables the quantification of materials by separating their attenuation characteristics into the different contributions of photoelectric absorption and Compton scatter [8, 12–15]. Nowadays, DECT is clinically used for diagnostic purposes such as classification of uric acid versus non-uric acid urinary stones or to quantify contrast media (CM) uptake, e.g., the local concentration of iodine in liver tissue [9, 16–19]. Experimental studies also suggest the use of non-approved CM with spectral properties that could be utilized for CTA or cancer theranostics.

A particular therapy that potentially would benefit from DECT quantification is selective internal radiation therapy (SIRT) with beta-emitting radioactive holmium-166 microspheres (^166^HoMS), which are currently used for radioembolization of liver tumors and intratumoral injection in solid malignancies [20–23]. In vivo dosimetry after therapy application, needed to verify the treatment success, can be performed based on SPECT imaging utilizing the holmium-166 gamma radiation or based on magnetic resonance imaging (MRI) utilizing the paramagnetic properties of holmium. Both modalities have shown their possibilities for application in radioembolization therapy; however, for intratumoral therapy, their use might be hampered by resolution limitations or detection limits [23–26].

In intratumoral therapy, high concentrations of microspheres are injected at several locations in the tumor to achieve proper dose levels for the entire tumor. This requires high-resolution dosimetry that cannot be achieved by SPECT and quantification of high holmium concentrations which is challenging for MRI.

Conventional CT for quantification of ^166^HoMS, utilizing the high attenuation coefficient of holmium, has previously been explored [24, 25]. CT in general would allow for fast ^166^HoMS quantification of high local concentrations with high spatial resolution and thus accurate local dosimetry at low cost. Although a clear relation was demonstrated between local holmium concentrations and CT signal, discrimination of holmium from, e.g., bone, calcified arteries, or iodinated CM, was found to be difficult [24].

It is expected that DECT can improve the previously identified limitations of conventional CT by utilizing the spectral information that DECT offers combined with the presence of a k-edge at 56 keV that holmium expresses, leading to a sudden increase in X-ray attenuation at that energy.

The objective of this study was to demonstrate the feasibility of DECT-based quantification of non-radioactive HoMS by means of phantom and measurements of rabbit cadavers.

## Materials and methods

In this study, the following experiments were performed: Firstly, a spectral calibration was performed to determine the tube voltage combination–dependent and object size–specific spectral properties of the investigated material. Secondly, contrast media quantification measurements were performed to define detection limits and accuracy. Finally, the feasibility of holmium quantification was tested in a pilot study performed in situ in VX-2 tumor-bearing rabbits. Since only a very small fraction of holmium-165 is converted to holmium-166 after neutron activation during production (approximately 0.001% [23]), no difference is expected in X-ray attenuation between radioactive and non-radioactive microspheres. Therefore, all experiments were performed using ^165^HoMS to avoid unnecessary radiation risks.

### Spectral calibration

Spectral calibration is required to depict an energy-dependent and material-dependent material vector [12]. For this purpose, a dedicated spectral calibration phantom (QRM GmbH) was scanned using a third-generation dual-energy CT scanner (SOMATOM Force, Siemens Healthcare GmbH). The scans were performed using a dual-energy abdomen protocol; collimation 2 × 64 × 0.6 mm with a flying focal spot in the z-direction, rotation time 0.5 s/rot, and a pitch of 0.6. The images were reconstructed using a weighted filtered backprojection algorithm [27] providing linear handling of contrast, noise, and spatial resolution in order to preserve purely quantitative results. All images were reconstructed with a slice thickness of 1.5 mm, an increment of 1.0 mm, a quantitative medium smooth reconstruction kernel (Qr44f) used for routine reconstruction of DECT images (50% value of the modulation transfer function: ρ_50_ = 4.62 lp/cm, no edge enhancement), and a reconstruction field of view (FOV) of 175 mm. The phantom consists of a 10-cm-wide cylinder comprising a synthetic material exhibiting liquid water-equivalent CT values. Two 2-cm-diameter syringes (Omnifix Solo, B. Braun) were located in the phantom’s center and 3 cm horizontally offset, respectively. The centric syringe was filled with holmium (III) chloride hexahydrate (Metal Rare Earth Limited (Holmium content 41.3–45.5% (w/w)) suspended in distilled water (Fresenius SE & Co KGaA). Titration of the holmium chloride revealed a 42.0% holmium content resulting in a final concentration of 10.5 mg/mL. The known concentration served as a reference to facilitate quantitative measurements. The offset syringe contained pure water as a reference to the phantom’s original material. In the spectral calibration, the following effects were investigated using an automatic exposure control (CARE, Dose4D, Siemens Healthcare GmbH), the effect of X-ray tube voltage combinations and size-dependent spectral effects (Table 1). Measurements were performed using different X-ray tube voltage combinations: 70/150 Sn kV, 80/150 Sn kV, 100/150 Sn kV, and 80/140 kV, whereas *Sn* denotes the utilization of a 0.6-mm-thick tin pre-filtration used for increased spectral separation [6]. To investigate size-dependent spectral effects, additional extension rings were used to expand the 10-cm-wide phantom to 15, 20, 25, 30, or 35 cm [7, 26, 28].

**Table 1:** Effective tube current-time products regarding the different phantom sizes and all tube voltage combinations as investigated in the calibration measurements

Tube voltages (system A/system B) in (kV)	Tube current time products for different phantom sizes in (mAs)					
	10 cm	15 cm	20 cm	25 cm	30 cm	35 cm
70/150 Sn	16 / 16	18 / 16	37 / 22	80 / 36	180 / 16	410 / 95
80/150 Sn	16 / 16	16 / 16	23 / 22	45 / 36	95 / 60	202 / 95
100/150 Sn	16 / 16	20 / 16	34 / 22	60 / 36	109 / 60	195 / 95
80/140	16 / 16	16 / 16	16 / 16	30 / 16	65 / 16	142 / 24

### Material decomposition algorithm

**Fig. 1:**
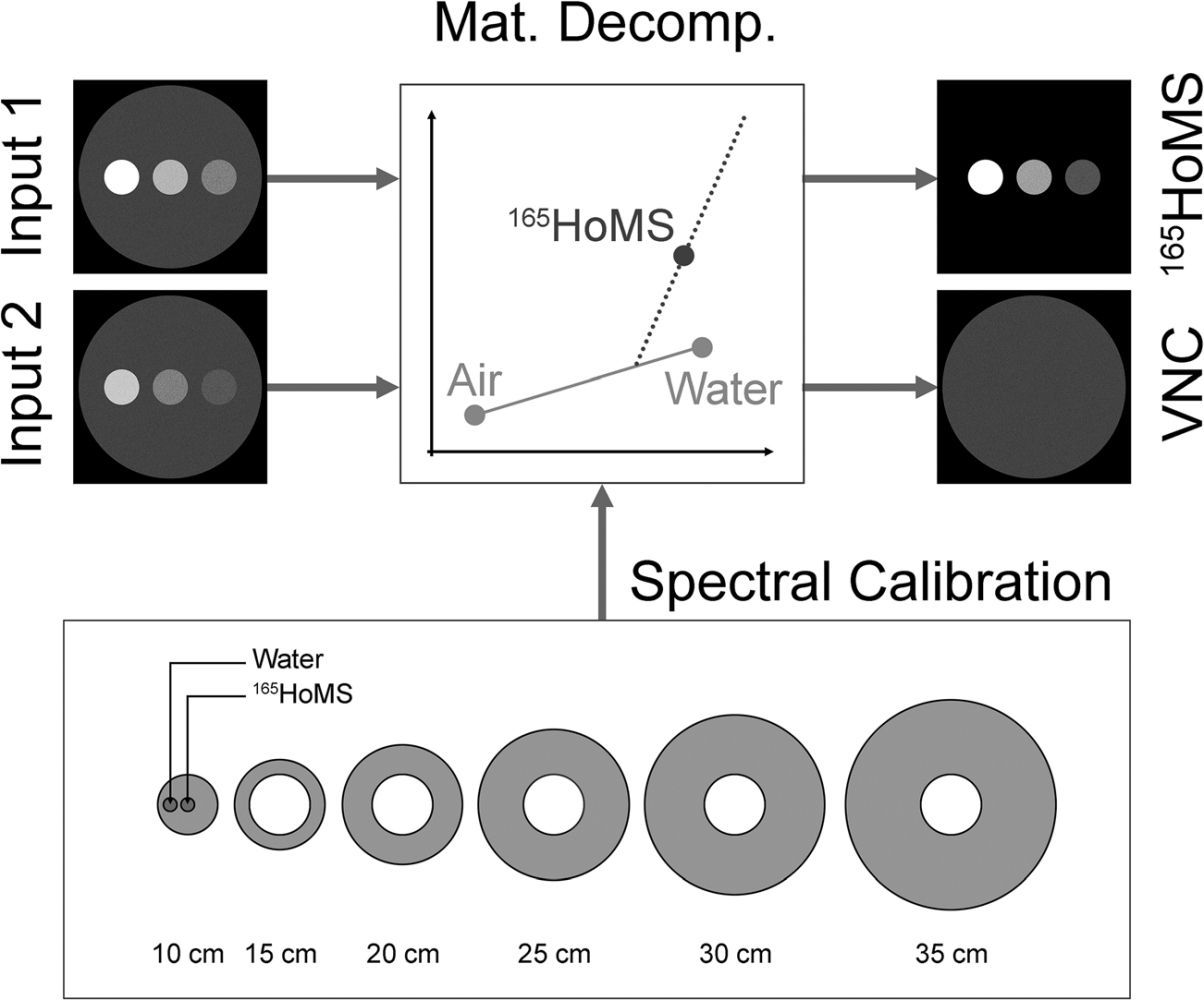
Spectral calibration scans are used to generate energy-dependent and material-dependent material vectors as a prerequisite for the image-based material decomposition. Therefore, the X-ray absorption of holmium on the applied X-ray spectra was investigated in relation to the size of the object (using 10–35-cm extension rings). Based on two different energy input images, inputs 1 and 2, the material decomposition can be calculated and results in material images constituting of local concentrations of ^165^HoMS, as well as virtual non-contrast images (VNC)

The relationship of the material-specific CT values of corresponding spectral CT acquisitions allows the determination of material vectors unique for the used tube voltages. The used image-based material separation algorithm (syngo.via, VB30A, Siemens Healthcare GmbH) relies on a base transformation and a projection of every measured set of CT values to preselected orthogonalized and linearly independent base vectors [8, 29]. In our study, air (− 1000/− 1000 HU for the low-kV and high-kV image, respectively), water (0/0 HU) and ^165^HoMS were selected as base materials. Decomposition into these three materials enables the generation of a material images that constitute local concentrations of ^165^HoMS, as well as virtual non-contrast images, mimicking a native scan where no CM was applied (Fig. 1).

### Contrast media quantification measurements

A serial dilution of HoMS (0.125–10.0 mg holmium/mL) was examined to test linearity with various concentrations and the detection limit. The concentration inserts were made of a batch of ^165^HoMS with a holmium mass percentage of 18.8% (QuiremSpheres, Quirem Medical B.V.). The microspheres were suspended in an injection solution containing 116 mmol phosphate-buffered saline (pH 7.2, 77.0 mmol disodium phosphate dibasic dehydrate, Merck Millipore, and 39.0 mmol sodium phosphate monobasic anhydrous, Sigma Aldrich) with polyoxyethylene-polyoxypropylene block copolymer (Pluronic F-68, Sigma-Aldrich, Chemie B.V.) 2% weight per volume solution. To prevent precipitation of ^165^HoMS due to their weight (1.4 g/mL), an agar solution (MP Agar, Roche Diagnostics) was added. The agar solution was heated to 90 °C for 10 min, resulting in a transparent fluid. The ^165^HoMS suspension and agar were mixed in a rising concentration of ^165^HoMS and filled into syringes for the spectral calibration measurements and into 5 -mL Eppendorf tubes for the quantitative comparison of measured and known concentration in the CT images. Once cooled to room temperature, the agar became solid.

The experimental setup consisted of a 20-cm-wide (real) water phantom that allowed the positioning of multiple tubes. The solutions were scanned in two groups (0.125–1.0 mg/mL and 2.0–10.0 mg/mL). The inserts were arranged in an equiangular fashion 5 cm distant from the phantom’s centerline.

In addition to the clinical dose (CARE Dose4D) measurements, a series of high-dose measurements were conducted (Table 2). With the first mode, the accuracy of ^165^HoMS quantification was assessed under clinical conditions, whereas the maximal tube current allows the determination of a contrast agent in a low noise situation. After the image-based material decomposition, the images were quantitatively assessed and ^16%^HoMS concentrations were compared with the known concentrations.

**Table 2:** Overview of the effective current, Q_ref_, and radiation dose for all applied tube voltage combinations for the quantification measurements

Tube voltages (system A/system B) in (kV)	Tube current determination	Effective current in (mAs)	Q_ref_ in (mAs)	Dose (CTDI32) in (mGy)
70/150 Sn	CARE Dose4D	212 / 55	380 / 95	4.36
	High Dose	1083 / 271	-	21.92
80/150 Sn	CARE Dose4D	105 / 54	190 / 95	3.83
	High Dose	1083 / 542	-	38.95
100/150 Sn	CARE Dose4D	60 / 53	190 / 95	4.22
	High Dose	840 / 420	-	48.91
80/140	CARE Dose4D	73 / 18	132 / 24	3.40
	High Dose	997 / 181	-	39.24

Equation 1 describes a tolerance term (derived from [30]) discerning as an estimate error for material quantification. The two terms presume the CT value stability and spectral stability to be two independent types of errors. The tolerance scales with increased known concentration *C*_known_.

1 $$ T=\sqrt{{\left(0.5\frac{\mathrm{mg}}{\mathrm{mL}}\right)}^2+{\left(0.1\cdotp {C}_{\mathrm{known}}\right)}^2} $$

### Animal preparation

All experiments were performed in conduct with *The Netherlands Experiments on Animals Act (1977)* and *The European Convention for the Protection of Vertebrate Animals used for Experimental Purposes (Strasbourg, 18.III.1986)*. Approval was obtained from the Utrecht University Animal Experiments Committee (DEC 2011.III.08.080).

The VX-2 tumor model was described previously [28]. Six tumors were induced by subcutaneous injection of three ± 1 mm^3^ viable fragments of VX-2 carcinoma harvested from the donor rabbit. Next, these fragments were injected with 0.1–0.3 mL PBS into three adult female New Zealand White (NZW) rabbits weighting 3–4 kg. All tumor implantations were performed under analgesia with carprofen 4 mg/kg. During the animal experiments, sedation and analgesia were achieved with a mixture of 0.125 mg/kg dexdomitor and 15 mg/kg ketamine.

Subsequently, aliquots with various amounts of ^166^HoMS were administered intratumorally. The exact administered amount, regarding holmium mass (mg), was calculated by measuring the radioactivity of the syringes with ^166^HoMS before and after injection using a VDC-404 dose calibrator, Veenstra Instruments B.V. Measurements performed on the DECT are primarily based on ^165^Ho as only a fraction is converted. Animals were sacrificed and preserved in a shielded freezer to let the radioactive holmium decay prior to scanning with the dual-energy CT scanner. The average water equivalent diameter of the rabbit cadaver ranged from 15 to 20 cm, such that the general quantitative detection of the ^165^HoMS as from the prior phantom scans can be assumed.

### Statistical analysis

After applying the image-based material decomposition algorithm, the quantitative material images were evaluated for ^165^HoMS quantification. Descriptive statistics (mean concentration, *R*^2^, RMSE, absolute and relative deviation) of the image-based material decomposition measurements were compared with the known concentrations for all tube voltage combinations and dose settings.

## Results

### Spectral calibration

**Fig. 2:**
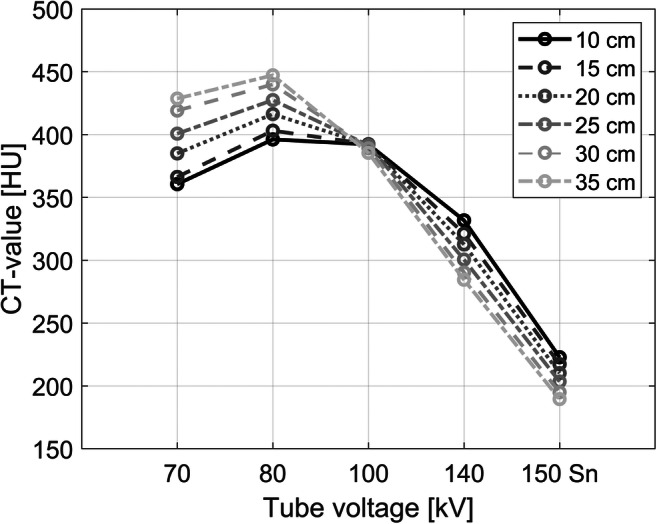
Measured CT values for 10.0 mg ^165^HoMS/mL with regard to different tube voltages and phantom sizes

Figure 2 shows that the X-ray absorption of holmium highly depends on the applied X-ray spectra and the size of the object. With an increased diameter, the mean energy of the X-ray spectrum shifts towards higher energies, which, for lighter atoms (presuming no k-edge in the applied X-ray spectrum), results in a decrease of X-ray attenuation and therefore lower CT values. An increased attenuation and an enhanced CT value are observed when the mean energy of an X-ray spectrum and its spectral barycenter moves closely beyond the k-edge of a material, where the photoelectric effect becomes dominant. The further the energies shift, the more the measured CT values decrease. For all investigated energies, both effects, the decreased X-ray attenuation due to increased object size and the simultaneously mean energy shift towards the k-edge of the material (56 keV for Holmium), result in an increase of total X-ray attenuation at 70 and 80 kV, but eventually to a decrease in absorption at 100–150 Sn kV (Fig. 2).

### Contrast media quantification measurements

**Fig. 3:**
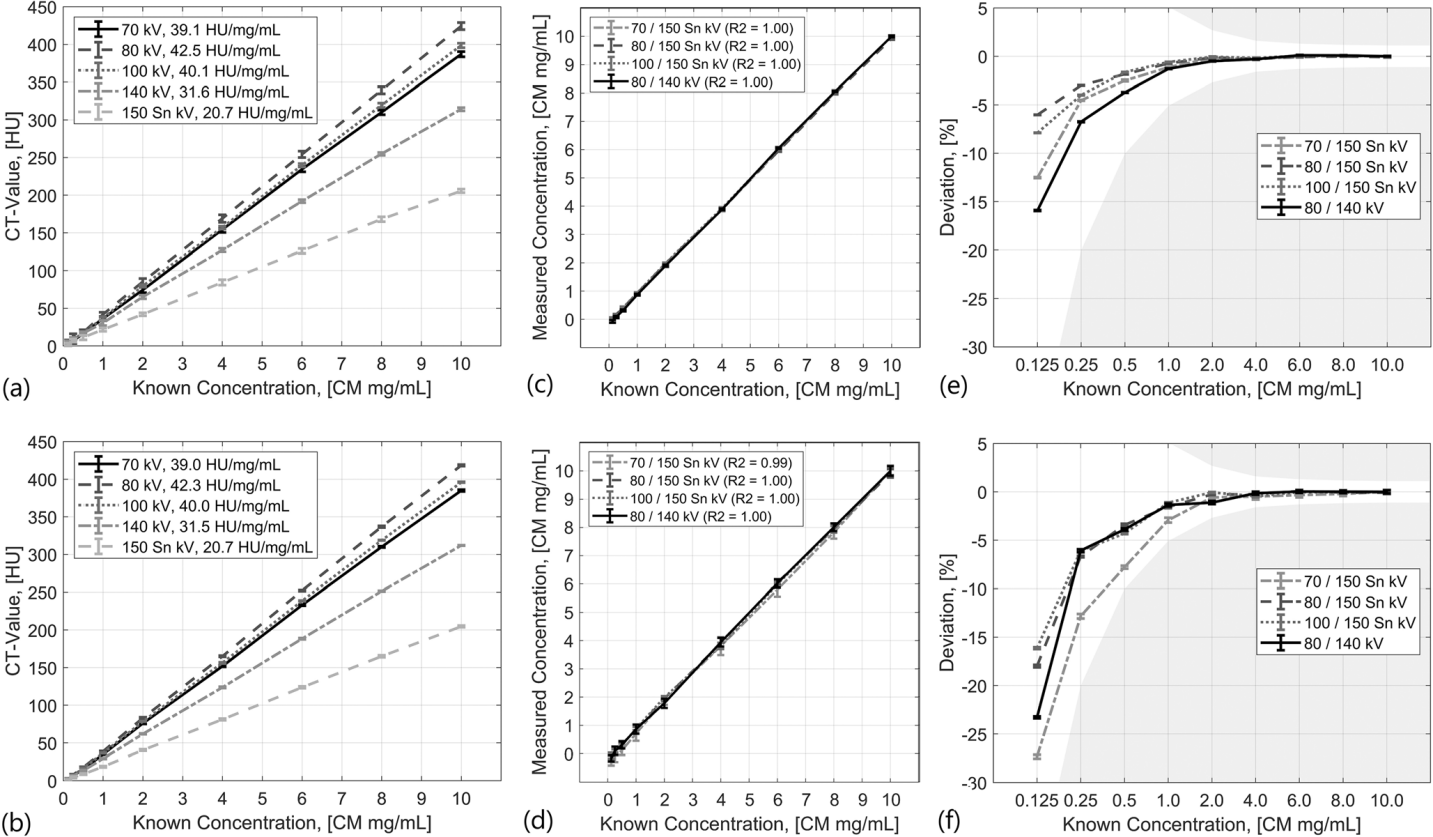
Slope and CT values for different ^165^HoMS mg/mL solutions in a 20-cm-wide water phantom. **a** High-dose measurements, (**b**) clinical situation with typical clinical radiation dose using automated exposure control. Accuracy of the measured concentrations compared against the known concentrations for high-dose measurements (**c**) and measurements using automated exposure control (**d**). Relative deviations of the measured concentrations compared against the known concentrations for high dose measurements (**e**) and measurements using automated exposure control (**f**). The grey area depicts the area where the given tolerance is exceeded

The CT values of the holmium solutions were identical for both dose levels and the image noise level scales square root to the radiation dose reduction (Fig. 3 a and b). Measured concentrations, in the phantom model, using CT quantification were linear (Table 3) and slightly lower than the known concentrations (Fig. 3 c and d). Figure 3 e and f show the increase of deviation with lower holmium concentrations. Given the introduced tolerance term, all quantifications down to 0.125 ^166^Ho-microspheres mg/mL were sufficient for all tube voltage combinations and for both clinical dose and high doses. The highest accuracy was observed using 80/150 Sn kV, followed by 100/150 Sn, 70/150 Sn, and 80/140 kV tube voltage and high radiation dose.

**Table 3:** Linear fit parameters (*m* for slope, *b* for offset) and errors (*R*^2^ and root mean square error, RMSE) for the comparison of measured and known concentration with respect to different tube voltage combinations and tube currents

Tube current	Tube voltage combination	Fit parameters and errors			
		*m*	*b*	*R*^2^	RMSE
CARE Dose 4D	70 / 150 Sn	1.03	− 0.32	0.99	0.25
	80 / 150 Sn	1.01	− 0.16	1.00	0.12
	100 / 150 Sn	1.02	− 0.16	1.00	0.12
	80 / 140	1.03	− 0.21	1.00	0.15
High Dose	70 / 150 Sn	1.02	− 0.13	1.0	0.09
	80 / 150 Sn	1.00	− 0.07	1.0	0.07
	100 / 150 Sn	1.01	− 0.08	1.0	0.06
	80 / 140	1.02	− 0.17	1.0	0.12

**Fig. 4:**
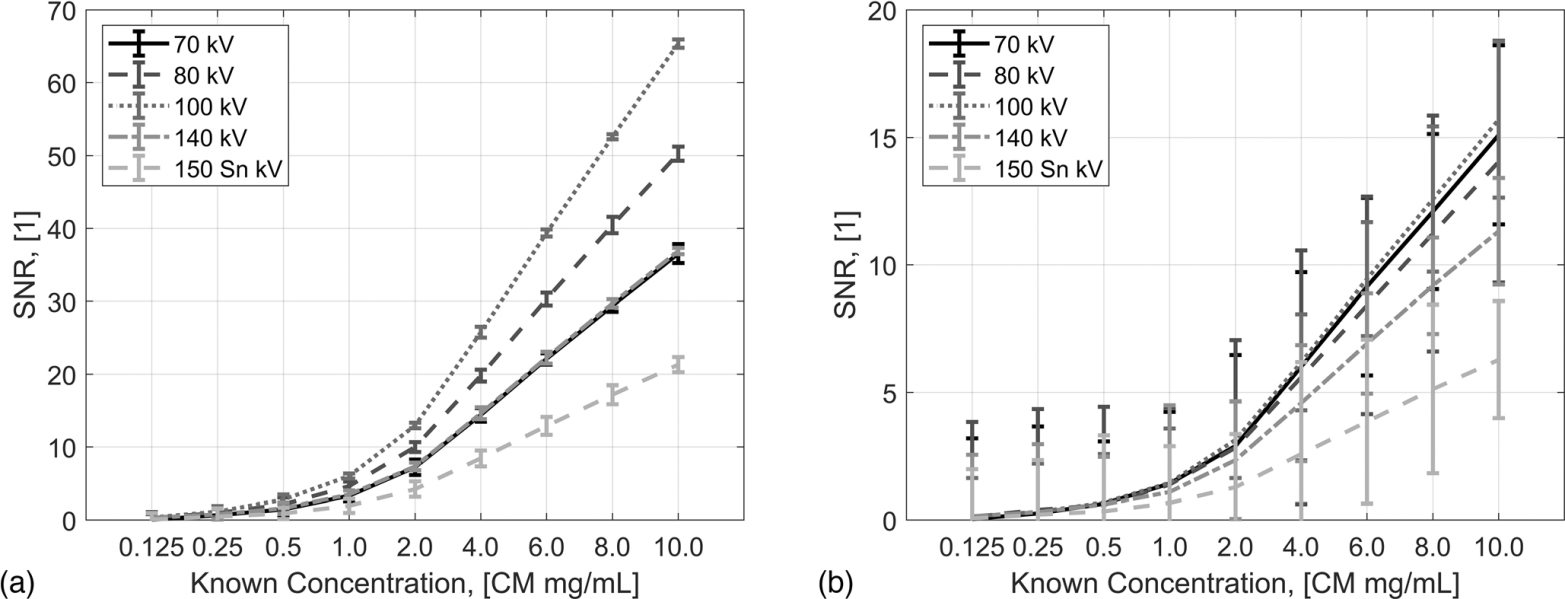
Measured SNRs for all investigated ^165^HoMS solutions using high radiation dose (**a**) and typical clinical radiation dose (**b**)

**Fig. 5:**
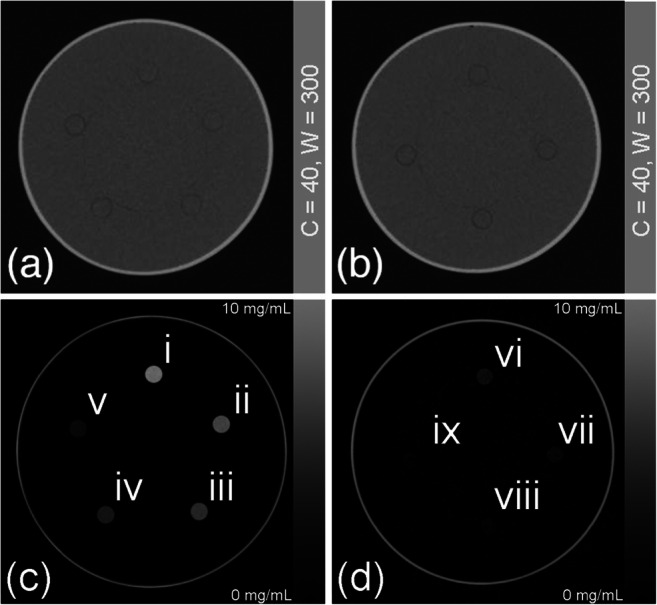
**a**, **b** VNC images, i.e., images where the ^165^HoMS is virtually removed. The material images (**c**, **d**) facilitate quantitative readout of the investigated ^165^HoMS concentrations: 10.0, 8.0, 6.0, 4.0, and 2.0 mg ^165^HoMS/mL; vi–ix show the results for 1.0, 0.5, 0.25, and 0.125 mg ^165^HoMS/mL

The measured CT values decreased with lower holmium concentrations, while the noise level remained the same for a tube voltage. This resulted in a factor 3–4 times lower SNR, e.g., from 65.4 to 15.7 for 80 kV with 10 mg/mL holmium (Fig. 4). The additional value of DECT material decomposition is shown in Fig. 5, illustrating the output of two separate material decompositions, virtual non-contrast images (Fig. 5 a and b) and the quantitatively measured mapping of holmium concentrations (Fig. 5 c and d).

### Animal experiment

**Fig. 6:**
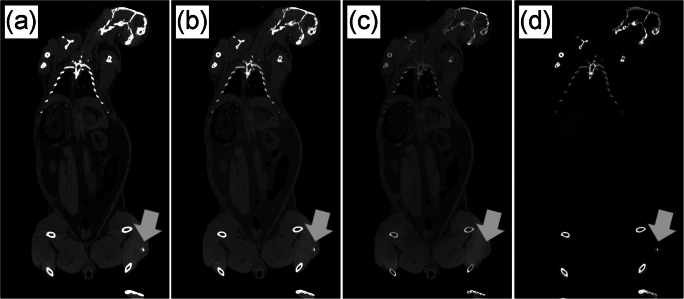
Coronary slice of a scanned rabbit cadaver acquired with 80 kV (**a**) and 150 Sn (kV) (center = 150 HU, window = 600 HU). After applying A VNC image (**c**) as well as a material-specific image (**d**) was calculated

Figure 6 depicts two acquired coronary slices of a scanned rabbit cadaver using 80 kV (a), 150 Sn kV (b), and a calculated VNC image (c) and a material-specific image (d). The material-specific image segments the holmium and soft tissue to show the exact location of the injected material with respect to the anatomy of the single animals (Fig. 6d). The injected and detected amount of ^165^HoMS corresponded well with relative deviation ranging between 1 and 11% (Table 4).

**Table 4:** List of injected amounts of ^165^HoMS and the detected amounts of ^165^HoMS after material decomposition and quantification of the animal measurements. The relative deviations (with reference to the injected amounts) are shown in the fourth column

Injections	Injected amount of ^165^HoMS (mg)	Detected amount of ^165^HoMS (mg)	Relative deviation (%)
1	5.61	6.3	11.4
2	3.34	3.0	− 11.7
3	5.96	5.5	− 7.7
4	12.59	12.8	1.7

## Discussion

In this article, we presented the first results of DECT-based quantification of holmium microspheres in phantoms and euthanized rabbits.

All measurements showed a linear relation between DECT-based holmium concentrations and actual holmium concentrations. This was observed for all investigated X-ray spectra. In the high-dose acquisitions, the accuracy per measurement is superior due to the reduced noise level compared with clinical (low) dose acquisitions. In the case of low concentrations of holmium, the reduced CT value can no longer be distinguished from the background signal anymore. This effect propagates through the applied material decomposition algorithm. Ultimately, the quality of the applied material separations based on the image data of the high-dose measurements outperformed the quality of the respective low-dose measurements.

The measured CT values of the ^165^HoMS solutions depended on the investigated phantom sizes. For lower energetic X-ray spectra (70 kV and 80 kV), the increased phantom size resulted in a shift of the mean X-ray spectra closer to the k-edge of holmium. The suddenly increased proportion of photoelectric absorption causes an increased total mass attenuation and therefore increased CT values. For higher energetic X-ray spectra (e.g., 140 kV or 150 Sn kV), the mean X-ray energy, as well as the spectral barycenter, moves away from the material’s k-edge, leading to reduced mass attenuation and decreased CT values. This effect is different for materials such as iodinated CM (k-edge at 33 keV) because the mean energies of typical X-ray spectra are already beyond that energy.

Material quantification for concentrations down to 0.125 mg/mL holmium was shown for all investigated tube voltage combinations and both evaluated dose levels. In the given experiment, a third-generation dual-source CT scanner was used. This scanner provides additional tube filtration to some tube voltages, which generally improves the spectral separation of dual-energy CT [6]. This option might not be available on all scanners, though. However, it was shown that a tube voltage combination of 80/140 kV could also be used to achieve an acceptable quantification of holmium solutions.

Measurements on three adult female NZW rabbits with subcutaneous VX-2 tumors, which were intratumorally injected with radioactive ^166^HoMS and imaged post-mortem, demonstrated DECT-based holmium quantification in a scenario that is much closer to clinical reality. Using parameters identical to the calibration scans, the measured amounts of holmium corresponded well with the known injected amount of holmium with a maximum deviation of 11.7%. Hypothetically, in a patient case where a tumor is injected with radioactive microspheres, the quantification is as follows: using the material decomposition method, the amount of holmium in mg per voxel can be calculated, with the known activity per mg at a certain timestamp given by the company in which the exact amount of activity at time-of-injection can be calculated. The last step is the conversion of an activity map (in MBq) to a dose map (in Gy) which can be realized by using a Monte Carlo–based dose point kernel, which is available in dose evaluation software packages. The dose point kernel takes into account the different radiation types (energies and particles), tissue characteristics, and the volume in which the energy is absorbed. This allows for the evaluation of the absorbed dose distribution on the tumor and can be related to follow-up to tumor-response.

The observation of the characteristic mass attenuation behavior with regard to the photon’s energies would facilitate k-edge-sensitive imaging for either dual- or even multi-energetic applications. This could potentially be used for simultaneous imaging of a patient’s vascularization and local accumulations of HoMS in tumorous regions within a single CT scan by virtually removing or quantifying one of the materials as shown in Figs. 5 and 6.

Impaired quality of the material separation that was found is largely a consequence of the experimental setup itself. The axial cross-sections of the investigated rabbits were rather elliptical, and the CT scanner did not correct for this shape with adaptive beam shaping. Additionally, the animals were partially frozen resulting in local changes of tissue densities leading to slightly deficiently determined soft tissue base material vectors. In our example, some tumor parts were not detected because of present partial volume effects as well as blooming artifacts caused by adjacent body materials (here: air and bone). In addition, the detection was suboptimal because third base materials are interpreted as a linear combination of the selected two base material vectors. This limitation could be overcome by extending the amount of applied or resolved X-ray spectra and by adding a third material to the equation, as it can be done using alternative spectral CT technologies such as photon counting detectors (PCD) [31, 32]. Investigating the quantification capability and robustness against clinically realistic artefacts that are per se inherent to a two material separation algorithm using a PCD-CT scanner is considered the next step in holmium microsphere imaging.

In conclusion, the first phantom and ex vivo holmium DECT data presented in this paper clearly show the feasibility to detect and quantify concentrations of holmium microspheres. This could lead potentially to the increased clinical utility of DECT imaging for dose verification during and after holmium microspheres internal radiation therapy.

## Abbreviations

^165^Ho: Holmium-165 isotope
^166^Ho: Holmium-166 isotope
CM: Contrast media
CS: Compton scatter
DECT: Dual-energy computed tomography
HoMS: Holmium microspheres
kV: Kilovolt
PE: Photoelectric effect
Sn: Tin (filter)
